# Clinicopathologic and prognostic significance of regulatory T cells in patients with hepatocellular carcinoma: a meta-analysis

**DOI:** 10.18632/oncotarget.17340

**Published:** 2017-04-21

**Authors:** Lejia Sun, Gang Xu, Wenjun Liao, Huayu Yang, Haifeng Xu, Shunda Du, Haitao Zhao, Xin Lu, Xinting Sang, Yilei Mao

**Affiliations:** ^1^ Department of Liver Surgery, Peking Union Medical College (PUMC) Hospital, PUMC and Chinese Academy of Medical Sciences, Beijing, 100730, China; ^2^ Department of General Surgery, Second Affiliated Hospital of Nanchang University, Nanchang, 330006, China

**Keywords:** regulatory T cells, FoxP3+, hepatocellular carcinoma, prognosis

## Abstract

The clinicopathologic and prognostic significance of regulatory T cells (Tregs) in patients with hepatocellular carcinoma (HCC) remains controversial. We performed a meta-analysis to resolve this issue. PubMed, Embase, Cochrane library, and the Web of Science were searched to identify eligible studies performed up to November 2016. A total of 3,854 HCC patients from 27 cohort studies were included. The meta-analysis revealed that high levels of Tregs were associated with poor overall survival (OS; HR = 1.95, *P* < 0.00001) and disease-free survival (DFS; HR = 1.82, *P* < 0.00001). However, the prognostic effect varied greatly according to the site of the Tregs. Higher intratumoral and peripheral blood levels of Tregs were associated with shorter OS and DFS, whereas a high peritumoral Tregs level was not associated with decreased OS and DFS. Trial design, therapy and method of detection had no effect on prognosis of Tregs. Moreover, the patients with high Tregs infiltration had multiple tumors, high AFP level, poor differentiation, later TNM stage, and vascular invasion. The present study demonstrates that high levels of intratumoral and peripheral blood Tregs predict multiple tumors, high AFP level, poor differentiation, later TNM stage, and vascular invasion and might be a promising prognostic factor in patients with HCC.

## INTRODUCTION

Hepatocellular carcinoma (HCC) is the fifth most common cancer and the third leading cause of cancer death worldwide. Half of these cases and deaths were estimated to occur in China [1, 2]. The current therapeutic options for HCC are limited to liver surgery and liver transplantation, but tumor recurrence following liver resection and liver transplantation for HCC is common and a major cause of death from this disease [3]. It is therefore necessary to study novel therapeutic strategies. The liver is considered a immune organ and immune escape is one of the mechanisms of hepatocarcinogenesis [4, 5]. The immunological microenvironment is very important for progression of HCC and regulatory T cells (Tregs) are in involved in the immunological microenvironment [6].

Tregs are a subgroup of CD4 +T cells characterized by expression of CD25, and forkhead or winged helix family of transcription factor P3 (FoxP3) is critical for the development and function of Tregs [7]. Tregs are important in maintaining self-tolerance and regulating immune responses in both physiologic and disease states. However, recent studies [8–10] have revealed that Tregs might play a role in tumor progression. Increased numbers of Tregs have been reported in peripheral blood and tumor tissues of patients with HCC and Tregs can impair CD8+ T-cell function in HCC, which is critical for immune evasion in liver cancer [11]. Several studies [12, 13] showed that injection of anti-CD25 antibody led to drastically enhanced antitumor immunity .Based on the above findings, Tregs seem to be a promising prognostic factor in patients with HCC and a high Tregs level has been reported to be correlated with poor outcomes in a number of publications [14–16]. However, whether Tregs have prognostic value in patients with HCC remains controversial.

Although two meta-analyses on prognosis have been conducted, they merely focused on intratumoral Tregs [17, 18] and the prognostic value of Tregs in peritumoral regions and peripheral blood was ignored. Moreover, whether Tregs infiltration is associated with clinicopathologic features in patients with HCC has not been analyzed systematically. For these reasons, we carried out this meta-analysis to derive a more precise estimation of the clinicopathologic and prognostic significance of regulatory T cells in patients with HCC.

## RESULTS

### Study selection

**Figure 1:**
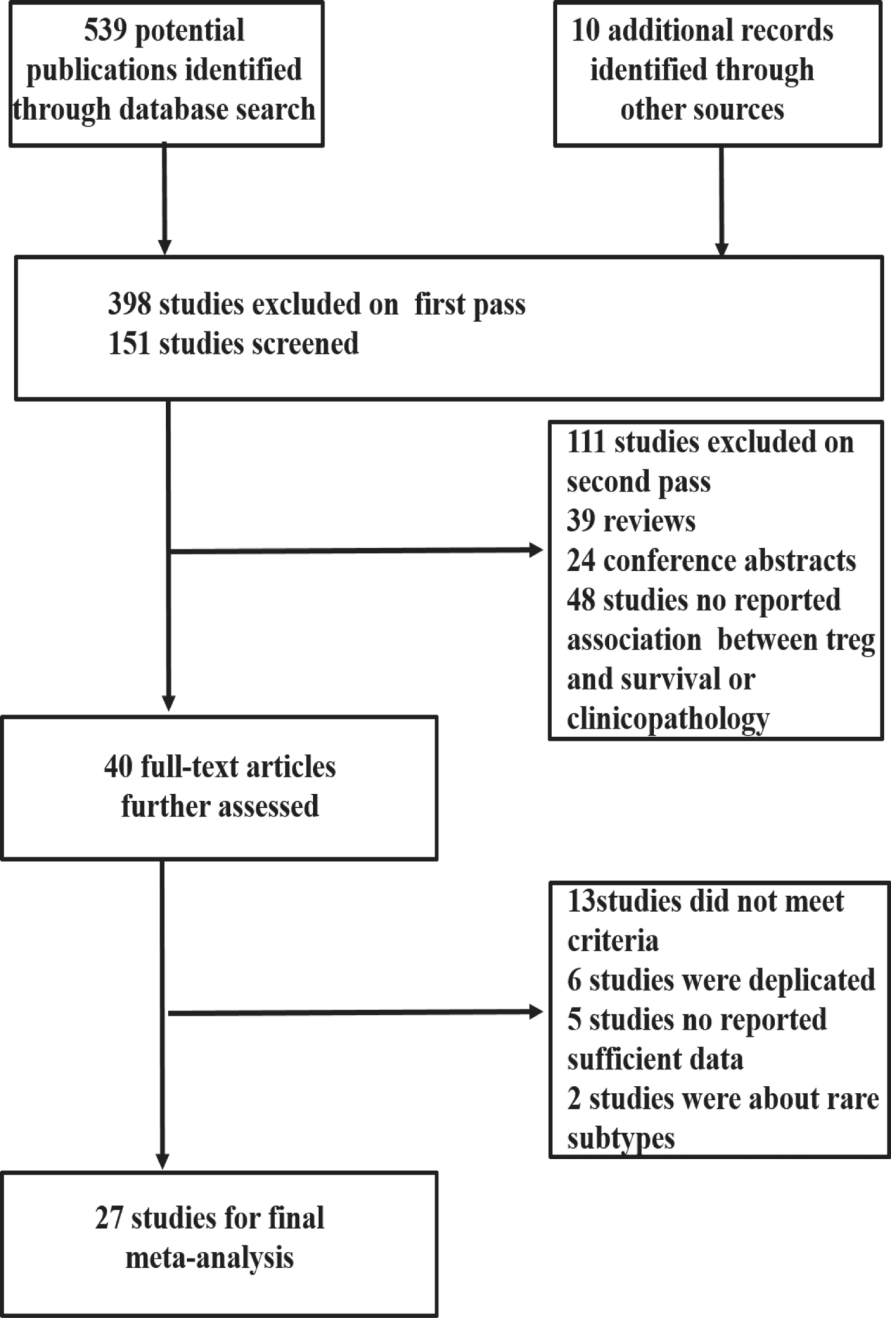
Flow diagram of the study selection process

Figure 1 shows our search and selection process. We identified a total of 549 articles in a systematic literature search. Forty potentially relevant studies were identified by reviewing the titles and abstracts. Of these, 13 studies were excluded because they did not meet the selection criteria. Finally, 27 studies were eligible for meta-analysis [14–16, 19–42].

### Characteristics of eligible studies

A total of 3,854 patients were included in the present study. The main features of each eligible study are summarized in Table 1. Four trial designs were prospective cohort studies and 23 were retrospective cohort studies. Most of the studies (23/27) were conducted in China, which was consistent with the high incidence of HCC in China. Twenty-one studies reported that surgery had been performed on patients. Patients in two studies had received transhepatic arterial chemotherapy and embolization (TACE). No prior treatment, cryoablation, and liver transplantation were each applied in one study.

**Table 1:** General characteristics of included studies

Author	Publication year	Country	No. of patients	Trial design	Therapy	Marker	Treg sites	Method	Cut -off	Follow up months, median (range)	Outcome measured	NOS
Esther et al.	2006	UK	69	RC	LT	FoxP3+	IT	IHC	0;1–2;3–10;≥ 10/5HPF	39.6 (2.4–202.8)	DFS	7
Fu et al.	2007	China	75	RC	Resection	CD4+CD25+FoxP3+	PB	FCM	≥ Mean	NR	OS	5
Kobayashi et al.	2007	Japan	147	RC	Resection	FoxP3+	IT	IHC	≥ Median	52.8 (0.5–169.1)	OS, DFS	6
Gao et al.	2007	China	302	RC	Resection	FoxP3+	IT	IHC	≥ Median	58 (2.0–109.0)	OS, DFS	7
Sasaki et al.	2008	Japan	164	RC	Resection	FoxP3+	IT, PT	IHC	≥ 14/10HPF	55.5 (2.0–184.0)	DFS	7
Gao et al.	2008	China	240	RC	Resection	FoxP3+	IT	IHC	Not applicable	16.0 (1.5–68.0)	OS, DFS	7
Ju et al.	2009	China	207	RC	Resection	FoxP3+	PT	IHC	≥ Median	27.9 (1.5–77.0)	OS, DFS	7
Zhou et al.	2009	China	121	RC	Resection	FoxP3+	IT	IHC	≥ Median	NR	OS, DFS	5
Ju et al.	2009	China	130	RC	Resection	FoxP3+	PT	IHC	≥ Mean	31.8 ± 1.7 (1.5–77.0)	OS, RFS	7
Wang et al.	2010	China	140	RC	Resection	FoxP3+	IT	IHC	Not applicable	NR	Not applicable	5
Lin et al.	2010	China	102	RC	Resection	FoxP3+	IT	IHC	≥ Mean	36.0 (1.0–84.0)	OS	7
Zhou et al.	2010	China	111	PC	Cryoablation	CD4+CD25+FoxP3+	PB	FCM	≥ Median	12	DFS	4
Chen et al.	2011	China	293	RC	Resection	FoxP3+	IT	IHC	≥ 6.6/HPF	NR	OS, DFS	5
Shen et al.	2011	China	76	PC	Resection	FoxP3+	IT	IHC	≥ 27/5HPF	12 (9.0–19.0)	OS, DFS	7
Chen et al.	2012	China	141	RC	Resection	FoxP3+	IT, PT	IHC	≥ Median	22.7 (2.0–70.3)	OS, DFS	7
Li et al.	2012	China	122	PC	TACE	CD4+CD25+CD127–	PB	FCM	≥ 6.7/HPF	NR	OS	6
Huang et al.	2012	China	55	RC	Resection	FoxP3+	IT, PT	IHC	≥ 10.8/HPF; ≥ 1.4/HPF	21 (2–49)	OS, DFS	7
Wang et al.	2012	China	137	RC	Resection, RFA, TAE	CD4+CD25+FoxP3+	IT, PB	IHC, FCM	≥ 14.55/HPF, ≥ Median	27.5 (2–49)	OS, DFS	7
Lin et al.	2013	China	245	RC	Resection	FoxP3+	IT	IHC	Not applicable	NR	OS, DFS	4
Huang et al.	2014	China	56	RC	Resection	FoxP3+	IT	IHC	≥ Median	36 (2–73)	OS, DFS	6
Li et al.	2014	China	264	RC	TACE	CD4+CD25+FoxP3+	PB	FCM	≥ Mean	NR	OS	6
Zhou et al.	2016	China	49	RC	Resection	CD4+CD25+FoxP3+	PB	FCM	≥ 5.07%	NR	DFS	7
Wang et al.	2016	China	141	RC	Resection	CD4+FoxP3+	IT	IHC	Not applicable	NR	OS, DFS	4
Wang et al.	2016	US	64	PC	Resection	FoxP3+	IT	PCR	Not applicable	48.2	OS	5
Tu et al.	2016	China	57	RC	Resection	FoxP3+	IT	IHC	≥ 3.2/HPF	NR	OS	4
Fu et al.	2016	China	348	RC	Resection	FoxP3+	IT	IHC	Not applicable	53.4 (1.5–61.3)	DFS	6
Cai et al.	2016	China	324	RC	Resection	FoxP3+	IT, PT	IHC	Not applicable	61.03 (2–82.33)	OS, DFS	6

LT, liver transplantation; IHC, immunohistochemistry; FCM, flow cytometry; WB, western blot; qRT-PCR, quantitative real-time polymerase chain reaction; PCR, polymerase chain reaction; PC, prospective cohort; RC, retrospective cohort

IT, intratumoral; PT, peritumoral; PB, peripheral blood; NR, not reported; OS: overall survival; DFS: disease-free survival; HPF, high-power field median/mean value was defined as the ratio of corresponding tumor islet and stroma counts.

Among the 27 studies, 26 reported OS and/or DFS and 15 presented the connection between clinicopathologic features and Tregs. Only one reported neither OS nor DFS, but presented clinicopathologic features. Tregs detected in studies were mainly intratumoral (20/27). In addition, six studies reported an association between Tregs in peripheral blood and prognosis, and six reported an association between Tregs in peritumoral sites and prognosis. Five studies reported the association between Tregs in two kinds of specimens at the same time and prognosis. The most commonly used test methods for Tregs were immunohistochemistry (IHC) and flow cytometry (FCM). One study used quantitative real time polymerase chain reaction (qRT-PCR). Tregs markers referred to CD4+CD25+ and FoxP3+ alone or in different combinations. One study used CD4+CD25+CD127− as the marker of Tregs. The mean follow-up ranged from 12 months to 61.03 months. The cutoff points of high Tregs infiltration were heterogeneous and half of the studies used the median number of Tregs as the cutoff point. Eighteen of the included studies had a quality score ≥ 6.

### Prognostic effect of tregs on survival

**Figure 2:**
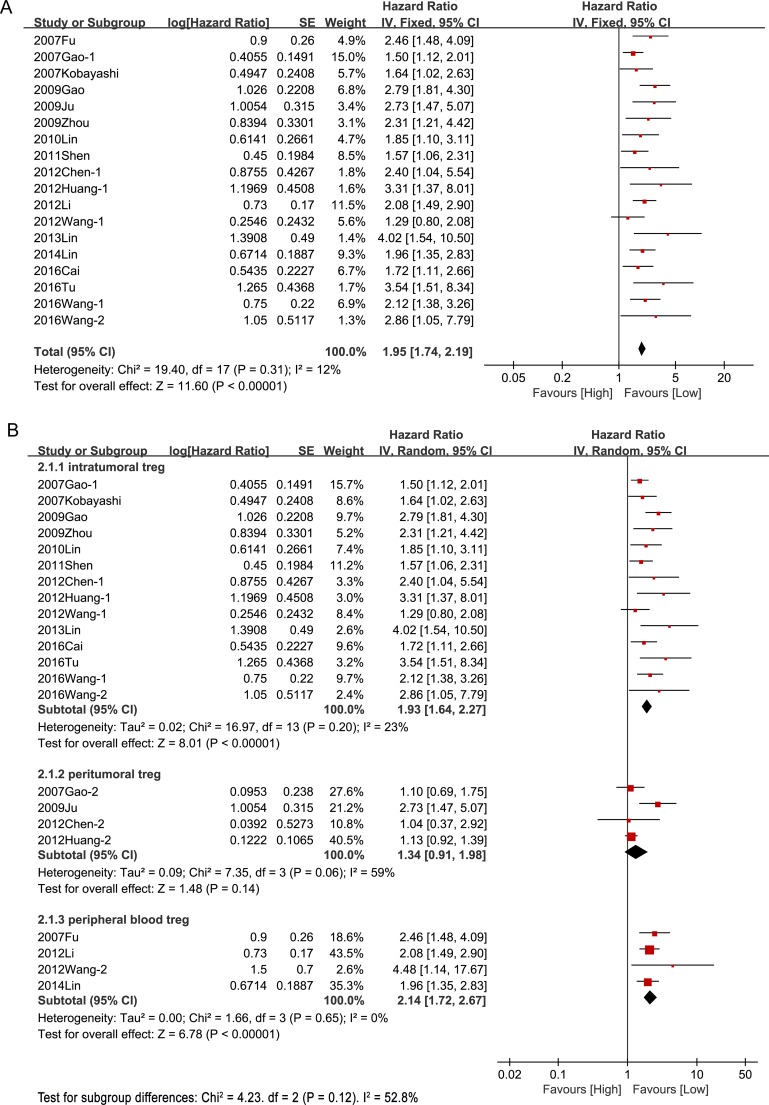
Prognostic effect of Tregs on overall survival (**A**) Prognostic effect of Tregs on overall survival without consideration of Tregs site; (**B**) Prognostic effect of Tregs in different sites on overall survival.

Eighteen studies with a total of 3,091 patients reported OS. Without considering the site of Tregs, the meta-analysis of all these studies confirmed a significant association between Tregs and survival—high Tregs level was associated with a significantly lower OS in patients with HCC (HR = 1.95, 95% CI [1.74, 2.19], *P* < 0.00001) and there was no significant heterogeneity between studies (I^2^ = 15%, *P* = 0.27) (Figure 2A). However, it was interesting that Tregs in different sites did not show the same outcomes. Higher Tregs levels in intratumoral tissue and peripheral blood were associated with shorter OS (intratumoral: HR = 1.93, 95% CI [1.64, 2.27], *P* < 0.00001; peripheral blood: HR = 2.14, 95%CI [1.72, 2.67], *P* < 0.00001), whereas higher Tregs level in peritumoral sites was not associated with decreased OS (HR = 1.34, 95%CI [0.91, 1.98], *P* = 0.14) (Figure 2B).

**Figure 3:**
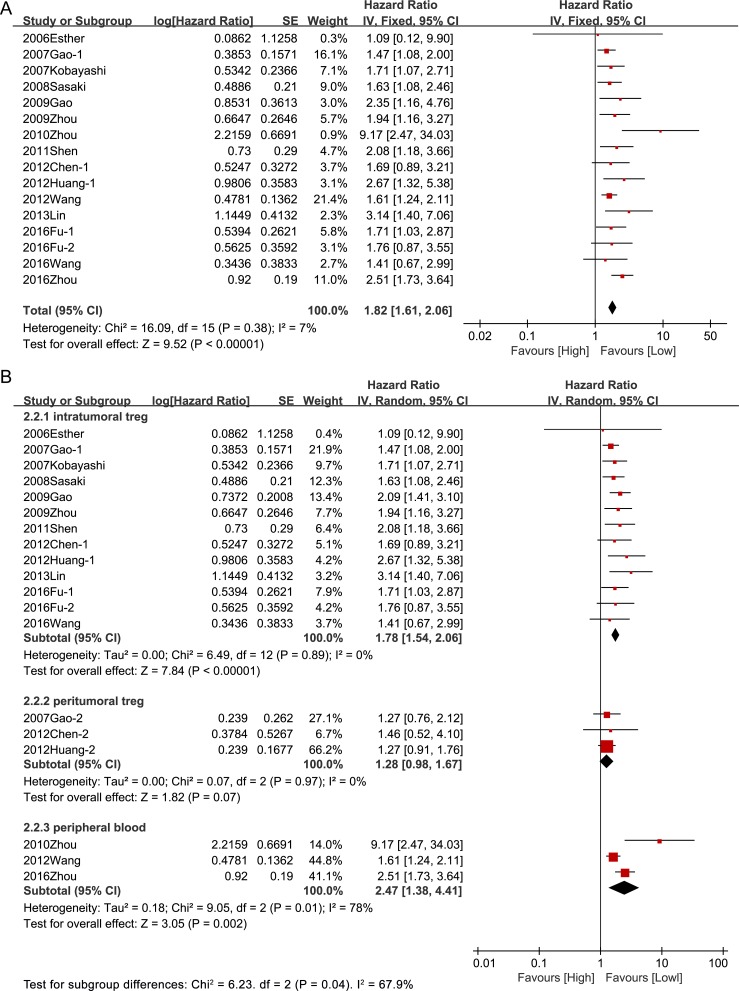
Prognostic effect of Tregs on disease-free survival (**A**) Prognostic effect of Tregs on disease-free survival without consideration of Tregs site ;(**B**) Prognostic effect of Tregs in different sites on disease-free survival.

Hazard ratios for DFS were available in 15 studies including 2,346 patients. The pooled result indicated that high Tregs level was associated with significantly poorer DFS in patients with HCC (HR = 1.82, 95% CI [1.61, 2.06], *P* < 0.00001) and no significant heterogeneity was observed between studies (I^2^ = 7%, *P* = 0.38) (Figure 3A). In line with the outcome of OS, higher Tregs levels in intratumoral sites and peripheral blood were associated with decreased DFS (intratumoral: HR = 1.78, 95% CI [1.54, 2.06], *P* < 0.00001; peripheral blood: HR = 2.47, 95%CI [1.38, 4.41], *P* = 0.002), but higher Tregs level in peritumoral sites was not associated with poor DFS (HR = 1.28, 95%CI [0.98, 1.67], *P* = 0.14) (Figure 3B).

A total of four studies were included for the meta-analysis of the association between peritumoral Tregs and prognosis. Peritumoral tissues were defined as liver tissues adjacent to the tumor within 10 mm without part of the tumor tissues in three studies and one study defined peritumoral tissues as liver tissues adjacent to the tumor beyond 20 mm.

**Figure 4:**
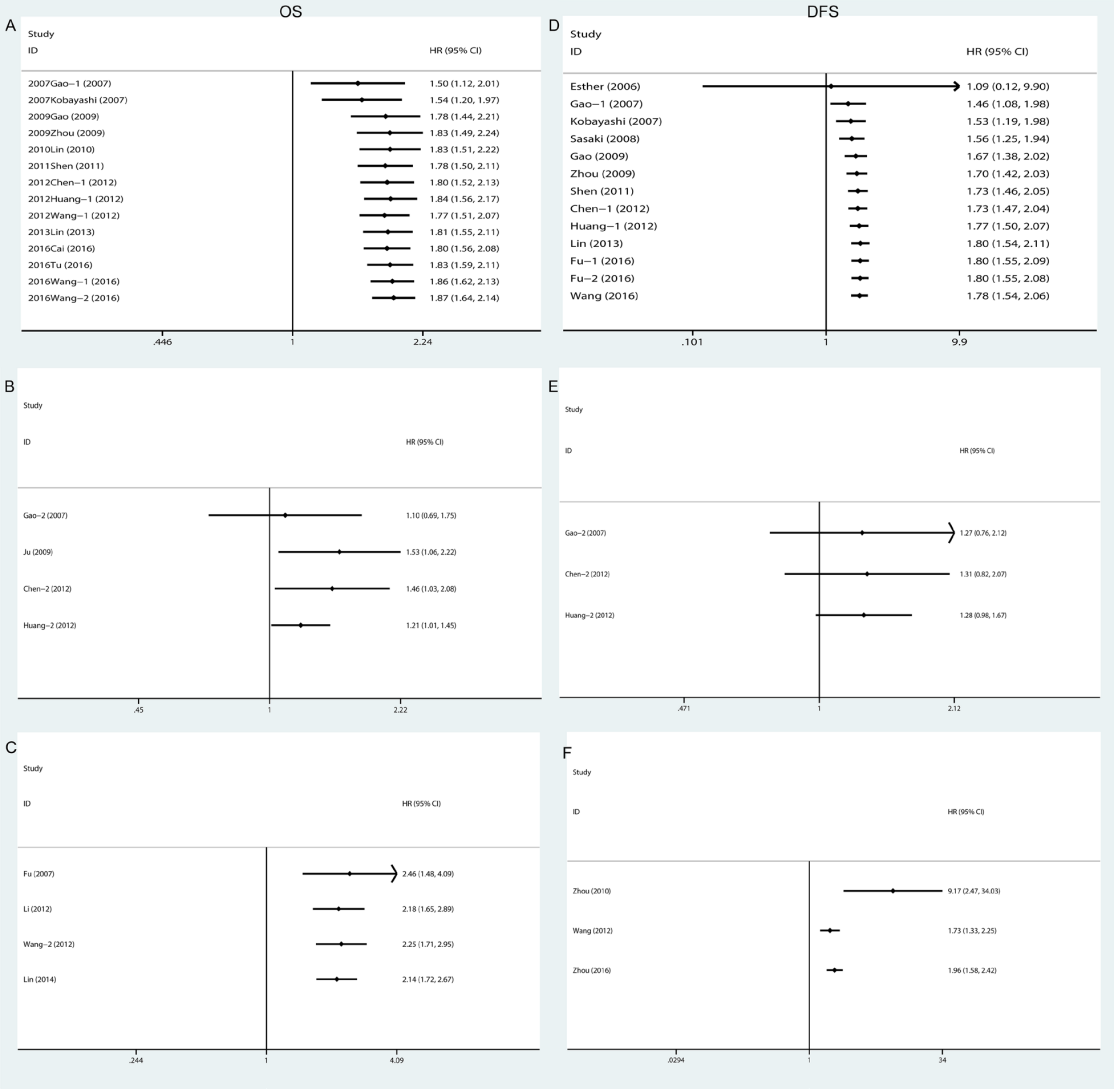
Cumulative meta-analysis of the association between Tregs and prognosis OS: (**A**) Intratumoral Tregs; (**B**) Peritumoral Tregs; (**C**) Peripheral blood Tregs; DFS: (**D**) Intratumoral Tregs; (**E**) Peritumoral Tregs; (**F**) Peripheral blood Tregs. Overall survival, OS; Disease-free survival, DFS.

The cumulative meta-analysis indicated that the results of the association between intratumoral Tregs and OS (Figure 4A) and DFS (Figure 4D) got more and more stable and the confidence interval got narrowed since the Gao's research in 2009. It is convinced that intratumoral Tregs were associated with poor prognostic for HCC, but the results of prognostic effects of Tregs in peritumoral regions and peripheral blood were not stable (Figure 4B, 4C, 4E, 4F).

### Subgroup analyses of the prognostic effect of tregs

Subgroup analyses were carried out to investigate potential sources of heterogeneity between studies and to assess whether conclusions were sensitive to restriction to subgroups that might have different prognostic effects. The results were summarized in Table 2. The First, we examined whether the therapy performed for patients affected estimates of the association between Tregs level and survival. Studies based on resection showed that high Tregs level was significantly associated with lower OS (HR = 2.03, 95% CI [1.76, 2.35], *P* < 0.00001) and DFS (HR = 1.89, 95% CI [1.66, 2.15], *P* < 0.00001), and pooled results from studies using TACE also showed that higher Tregs level was significantly associated with OS (HR = 2.02, 95% CI [1.58, 2.59], *P* < 0.00001). We also analyzed the trial design for Tregs level and found a significant effect on OS (prospective cohort: HR = 1.61, 95% CI [1.14, 2.26], *P* = 0.007; retrospective cohort: HR = 1.97, 95% CI [1.73, 2.24], *P* < 0.00001). However, the trial design had different effects on DFS (prospective cohort: HR = 3.86, 95% CI [0.92, 16.23], *P* = 0.07; retrospective cohort: HR = 1.82, 95% CI [1.16, 2.05], *P* < 0.00001). Besides, the subgroup analyses based on methods of detection showed that high Tregs level was associated with poor OS (immunohistochemistry: HR = 2.02, 95% CI [1.69, 2.42], *P* < 0.00001); flow cytometry: HR = 2.08, 95% CI [1.65, 2.63], *P* < 0.00001) and DFS (immunohistochemistry: HR = 1.81, 95% CI [1.58, 2.08], *P* < 0.00001; flow cytometry: HR = 2.47, 95% CI [1.38, 4.41], *P* = 0.002).

**Table 2:** Subgroup analyses of the prognostic effect of Tregs

Subgroup	Number of Studies	Test for association			Test for heterogeneity		
		HR	95% CI	*p*	Chi2	I^2^	*p*
**OS**							
**Therapy**							
Resection	13	2.03	[1.76, 2.35]	< 0.00001	14.08	15%	0.30
TACE	2	2.02	[1.58, 2.59]	< 0.00001	0.05	0%	0.44
**Trial design**							
Retrospective cohort	15	1.97	[173, 2.24]	< 0.00001	17.48	20%	0.23
Prospective cohort	3	1.61	[1.14, 2.26]	0.007	1.96	0%	0.38
**Method of detection**							
Immunohistochemistry	13	2.02	[1.69, 2.42]	< 0.00001	16.91	29%	0.15
Flow Cytometry	4	2.08	[1.65, 2.63]	< 0.00001	2.34	0%	0.51
PCR	1	2.86	[1.05, 7.79]	0.04	−	−	−
**DFS**							
**Therapy**							
Resection	14	1.89	[1.66, 2.15]	< 0.00001	8.88	0%	0.78
Liver transplantation	1	1.09	[0.12, 9.90]	0.94	−	−	−
Cryoablation	1	9.17	[2.47, 34.43]	0.0009	−	−	−
**Trial design**							
Retrospective cohort	15	1.82	[1.61, 2.05]	< 0.00001	9.96	0%	0.77
Prospective cohort	2	3.86	[0.92, 16.23]	0.07	4.15	76%	0.04
**Method of detection**							
Immunohistochemistry	14	1.81	[1.58, 2.08]	< 0.00001	6.51	0%	0.93
Flow Cytometry	3	2.47	[1.38, 4.41]	0.002	9.05	78%	0.01

OS, overall survival; DFS, disease-free survival; TACE, transhepatic arterial chemotherapy and embolization; PCR, polymerase chain reaction.

### Relationship between tregs and clinicopathologic characteristics

Fifteen studies reported the association between Tregs and clinicopathologic parameters. A total of 16 features were analyzed, including tumor size, AFP level, and vascular invasion. The information for various clinicopathologic parameters and their correlation with Tregs is summarized in Table 3. The results of meta-analysis demonstrated that patients with multiple tumors (OR = 0.73, 95% CI [0.56, 0.95], *P* = 0.02), high AFP level (OR = 0.66, 95% CI [0.52,0.84], *P* = 0.0007), poor differentiation (OR = 0.58, 95% CI [0.46.0.74], *P* < 0.0001), later TNM stage (OR = 0.64, 95% CI [0.49, 0.86], *P* = 0.003) and vascular invasion (OR = 2.2, 95% CI [1.50, 3.22], *P* < 0.0001) had high Tregs levels.

**Table 3:** Meta-analysis of reported clinicopathologic characteristics in the included studies

Parameters	Number of Studies	Test for association			Test for heterogeneity		
		OR	95% CI	*P*	Chi^2^	I^2^	*P*
Gender (Male vs.Female)	13	0.93	[0.74, 1.19]	0.58	14.82	19%	0.25
Age (≤ 50 vs. > 50)	3	1.12	[0.55, 2.26]	0.76	4.44	55%	0.11
Tumor size (≤ 5 cm vs. > 5 cm)	9	0.73	[0.47, 1.14]	0.17	20.73	66%	0.11
Tumor number (Single vs. Multiple)	9	0.73	[0.56, 0.95]	0.02	3.23	0%	0.86
AFP level (≤ 400 vs. 400 ng/mL)	6	0.66	[0.52, 0.84]	0.00	9.12	45%	0.10
ALT level (≤ 40 vs. 40 U/L)	3	1.15	[0.62, 2.12]	0.65	7.89	75%	0.02
Liver cirrhosis (Yes vs. No)	11	1.31	[0.80, 2.14]	0.28	28.79	65%	0.00
Vascular invasion (Yes vs. No)	10	2.20	[1.50, 3.22]	0.00	20.52	56%	0.01
Tumor encapsulation, (Presence vs. Absence)	6	1.00	[0.78, 1.29]	0.89	8.76	43%	0.12
TNM stage (I+II vs. III+IV)	6	0.64	[0.49, 0.86]	0.00	3.18	0%	0.67
Child-Pugh score (A vs. B+C)	7	1.16	[0.74, 1.81]	0.52	7.60	21%	0.27
Tumor differentiation (I+II vs III+IV)	7	0.58	[0.46, 0.74]	0.00	3.69	0%	0.72
PVTT (Yes vs. No)	4	1.43	[0.97, 2.10]	0.07	5.29	43%	0.15
History of hepatitis (Yes vs. No)	10	1.50	[0.83, 2,71]	0.18	35.64	75%	0.00
HBsAg (Positive vs. Negative)	5	0.78	[0.54, 1.12]	0.17	6.91	43%	0.14
HBeAg (Positive vs. Negative)	5	1.36	[0.86, 2.14]	0.19	1.62	0%	0.80

AFP, alpha fetoprotein; ALT, alanine transaminase; PVTT, portal vein tumor thrombus.

Controversies have existed regarding the correlation among tumor size and liver cirrhosis. We found that the incidence of Tregs in tumors of size >5 cm was higher than that in tumors ≤5 cm, but the difference did not reach statistical significance (OR = 0.73, 95 % CI [0.47, 1.14], *P* = 0.17). Likewise, patients with liver cirrhosis had higher Tregs levels than those without liver cirrhosis but without statistical significance (OR = 1.13, 95% CI [0.80,2.14], *P* = 0.28). Besides, the results of meta-analysis demonstrated no correlation between infiltration of Tregs and gender (OR = 0.93, 95% CI [0.74,1.19], *P* = 0.58), age (OR = 1.12, 95% CI [0.55,2.26], *P* = 0.76), tumor encapsulation (OR = 1.00, 95%CI [0.78,1.29], *P* = 0.89), Child-Pugh score (OR = 1.16, 95% CI: [0.74,1.81], *P* = 0.52), history of hepatitis (OR = 1.50, 95% CI [0.832, 71], *P* = 0.18), HBsAg (OR = 0.78, 95% CI [0.54, 1.12], *P* = 0.17), HBeAg (OR = 1.36, 95% CI [0.86, 2.14], *P* = 0.19), and portal vein tumor thrombus (OR = 1.43, 95% CI [0.97,2.10], *P* = 0.07).

### Sensitivity analyses and publication bias

**Figure 5:**
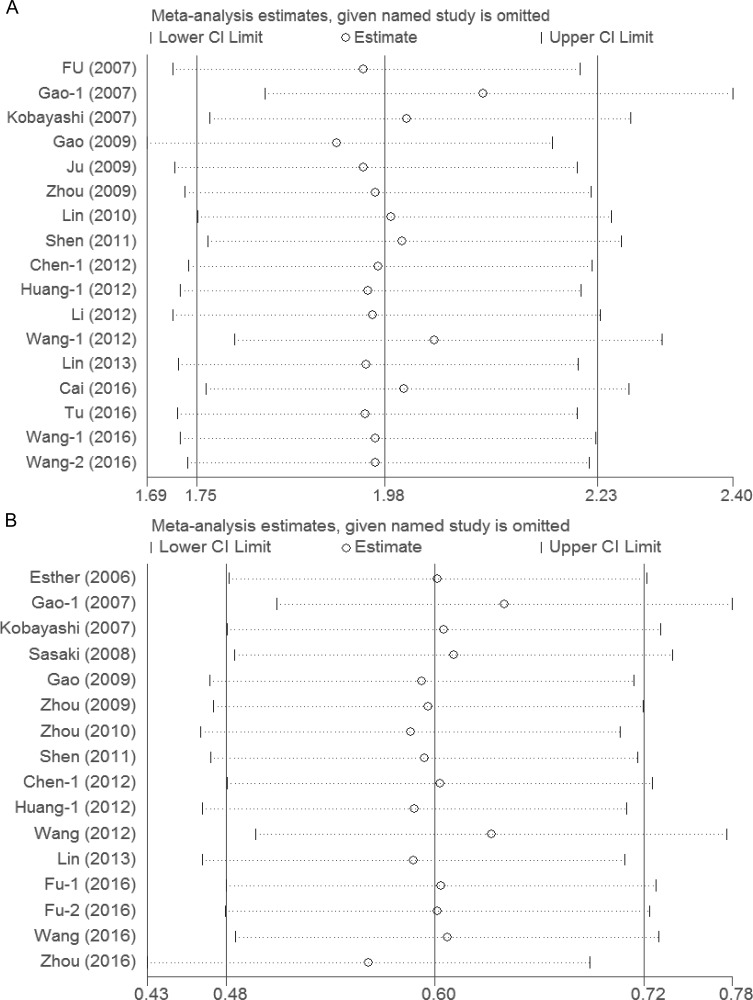
Sensitivity analyses of the association between Tregs and prognosis (**A**) Sensitivity analysis of the association between Tregs and overall survival; (**B**) Sensitivity analysis of the association between Tregs and disease-free survival.

**Figure 6:**
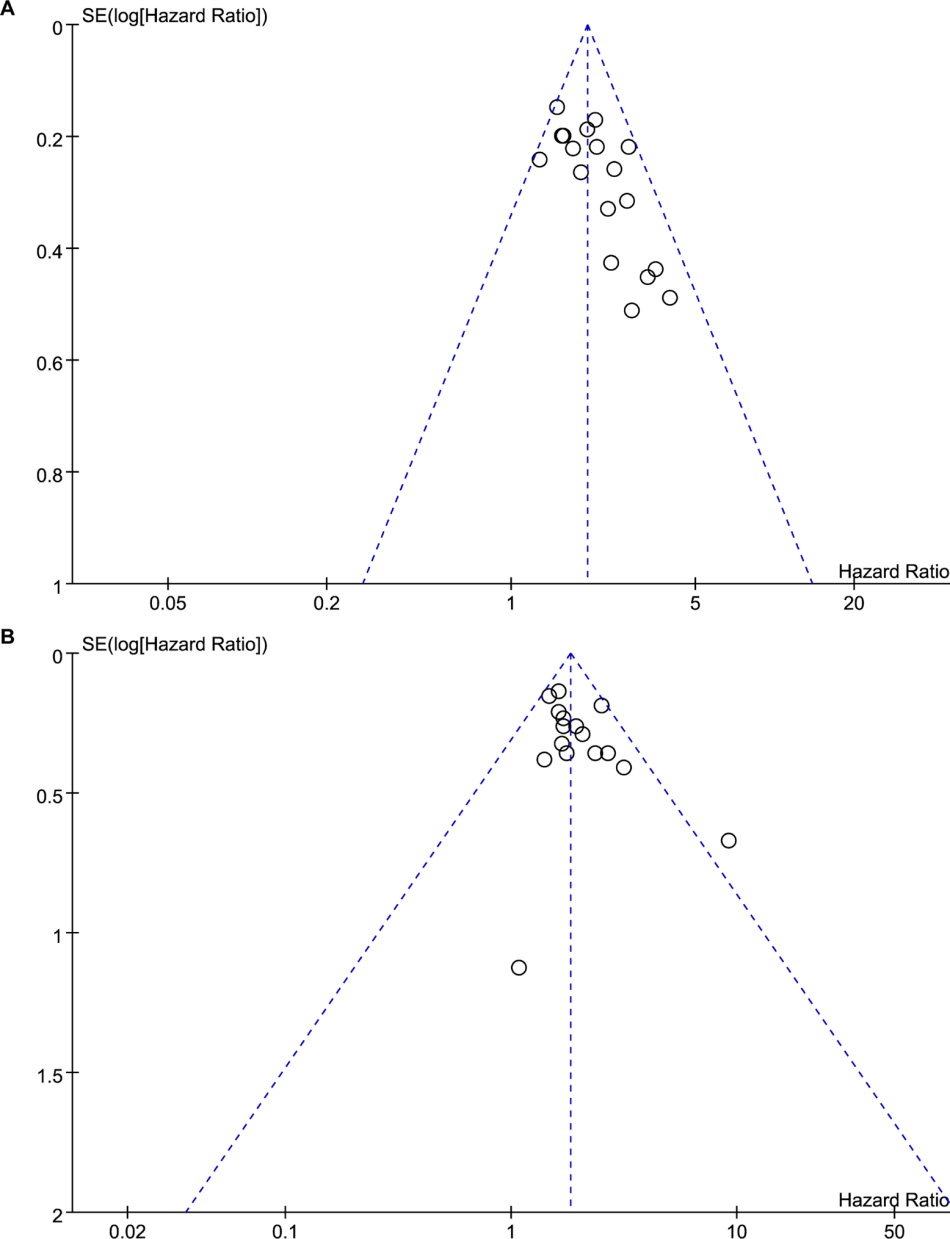
Funnel plots of the association between Tregs and prognosis (**A**) Funnel plot of the association between Tregs and overall survival; (**B**) Funnel plot of the association between Tregs and disease-free survival.

Sensitivity analyses showed that the association between Tregs and prognosis was robust (Figure 5). Funnel plot was performed to assess the publication bias of this meta-analysis. As shown in Figure 6, the distribution of the OS and DFS funnel plots were asymmetric, which indicated publication bias. In addition, there was some degree of publication bias in the studies on clinicopathology.

## DISCUSSION

High Tregs levels have different effects on prognosis in different kinds of cancer [18]. A high Tregs level was associated with poor prognosis in breast cancer [43] but with improved prognosis in colorectal cancer [44]. This study addressed the prognostic value of Tregs in HCC. Previous studies [17, 18] reported that high intratumoral Tregs levels were associated with poor prognosis in HCC. We included a larger number of studies in a meta-analysis and reached the same conclusion that a higher Tregs level was associated with significantly lower OS and DFS in patients with HCC without consideration of Tregs site. However, there was controversy over whether high Tregs levels in other sites, such as peripheral blood and peritumoral regions have the same effect on prognosis. We found that Tregs from different sites did not have the same effect on prognosis: increased Tregs levels in intratumoral sites and peripheral blood were associated with poorer OS whereas high Tregs levels in peritumoral regions had no association with OS. The same applied to DFS. Overall, a lower level of Tregs in intratumoral regions and peripheral blood might improve survival and reduce recurrence of HCC, and may be a promising therapeutic strategy for HCC.

Intratumoral Tregs are involved in tumor progression by inhibiting the function or maturation of antigen-presenting cells, destroying target cells, impeding the proliferation or activation of natural killer cells and effector T cells, causing metabolic disruption, secreting the immunosuppressive cytokines transforming growth factor beta (TGF-β) and IL-10, and expressing T lymphocyte–associated antigen 4 (CTLA-4) protein [45–48]. The mechanisms discussed above may lead to the association between Tregs and prognosis in HCC. Our finding that Tregs in different sites do not have the same effect on survival in HCC indicated that Tregs might play different roles in HCC according to their location. In particular, peritumoral Tregs did not appear to promote tumor progression. Wu et al.[49] reported a difference in quantity and phenotype among intratumoral, peritumoral, and peripheral blood Tregs; intratumoral Tregs had higher prevalence and more suppressive activity in HCC patients. There is accumulating evidence that FoxP3+ T cells in humans are heterogeneous in phenotype and function, consisting of suppressive and non-suppressive subpopulations. CD25^high^ FoxP3^high^CD45RA-cells are designated as effector or activated Treg cells, which are highly suppressive, and CD25^low^FoxP3^low^ CD45RA+ cells are designated as naive or resting Treg cells [50]. Perhaps, peritumoral Tregs might be mostly resting Treg cells, which are not involved in immune escape of HCC but important for avoiding autoimmunity. Some researches can be conducted to verify the assumption by flow cytometer and functional studies of peritumoral Tregs may help to explain the observed association with prognosis.

Our finding that Tregs in peripheral blood were associated with prognosis in a meta-analysis is meaningful because Tregs in peripheral blood can be detected easily and allow real-time monitoring compared with Tregs in the tumor. There are two reasons accounting for the prognostic effect of Tregs in peripheral blood. Tregs in peripheral blood are connected to intratumoral Tregs. HCC can recruit Tregs in peripheral blood and convert CD4^low^CD25^low^to CD4^high^CD25^high^ [28, 51]. Besides, immune escape of tumor occurs not only in local immunity but also in systemic immunity. Tregs in peripheral blood play an important role in immune tolerance and higher level of Tregs in peripheral blood could lead to immunosuppression of the whole immune system.

We also explored the clinicopathologic significance of Tregs. Zhao et al.[52] reported no association between Tregs and six clinicopathologic parameters of tumor number, AFP level, tumor size, TNM stage, HBV infection, and tumor capsule; however, there were only three articles that included each clinicopathologic parameter, which affected the reliability of the conclusion. Moreover, many other important parameters, such as tumor differentiation, vascular invasion, and portal vein tumor thrombus, have not been analyzed. Our study including a larger number of eligible studies showed that higher Tregs levels were associated with some clinicopathologic parameters, such as multiple tumors, higher AFP level, poor tumor differentiation, later TNM stage, and vascular invasion. This conclusion further supported the meta-analysis results on OS and DFS because patients with multiple tumors, high AFP level, poor differentiation, later TNM stage, and vascular invasion have poor prognosis [19–24].

Vascular invasion is associated with high Tregs level and Tregs can promote tumor metastasis; however, there is no research accounting for these associations. The present study showed that patients with portal vein tumor thrombus had high Tregs levels (although this was not significant), which was not consistent with vascular invasion. As more articles on vascular invasion were included in our analysis, the result was more stable and reliable. Although several studies showed that patients with high or low Tregs levels had liver cirrhosis [21, 23, 28], our present meta-analysis demonstrated no association between Tregs and liver cirrhosis. It is possible that Tregs play complicated roles in the progression of fibrosis in the liver. In brief, the association between Tregs level and several clinicopathologic parameters reconfirms that Tregs may be involved in HCC progression.

The present meta-analysis has several additional limitations that should be addressed. First, although we tried to identify all relevant data, potential publication bias was unavoidable. Several studies [40, 42, 53] reported no association between Tregs and survival, but without an available HR and/or 95% CI. Insignificant HRs for prognosis and ORs for clinicopathologic parameters are less likely to be reported in studies. We must therefore be cautious regarding our results. Second, heterogeneity could not be eliminated. There are many reasons for potential heterogeneity. Treg markers, Treg sites, follow-up time, and cut-off value were defined differently among studies, and the patients had received different treatments. However, we tried to reduce the impact of heterogeneity through subgroup analyses. Third, the number of included studies reporting Tregs in peripheral blood and peritumoral sites was relatively small. As there were insufficient eligible studies we did not conduct stratified analysis for the association between Tregs and clinicopathologic parameters according to Tregs site. Finally, the included studies are mostly retrospective studies and more high-quality prospective studies are needed to confirm our results.

Despite the limitations of our study, our meta-analysis is meaningful for demonstrating the correlation between prognosis and Tregs, especially Tregs in peripheral blood and peritumoral regions. Besides, this is the first comprehensive analysis of the association between clinicopathologic characteristics and Tregs in HCC. Sensitivity analyses revealed that the results were robust.

In conclusion, meta-analysis of available data suggests that a high Tregs level in intratumoral sites and peripheral blood was associated with OS and DFS, and may be a promising prognostic factor in patients with HCC. Patients with higher Tregs level tended to have multiple tumors, higher AFP level, poor differentiation, later TNM stage, and vascular invasion. Nevertheless, further well-designed clinical studies are needed to elucidate the exact relationship and the underlying mechanism.

## MATERIALS AND METHODS

The meta-analysis was performed in accordance with Preferred Reporting Items for Systematic Reviews and Meta-Analyses: the PRISMA Statement [54].

### Publication search strategy

We systematically searched PubMed, Embase, Cochrane library, and Web of Science up to November 2016, without restrictions on the region and language, for studies on the association between prognosis or clinicopathology and Tregs in patients with HCC. The following keywords were used when searching: (‘liver cancer’ or ‘hepatocellular carcinoma’), (‘regulatory T cells’ or ‘FoxP3′), and (‘prognosis’ or ‘clinicopathology’). We tried to identify additional pertinent studies by reviewing reference lists of the identified reports, reviews, meta-analyses, and other relevant publications. The “related articles” function was used at the same time to broaden the search.

### Inclusion and exclusion criteria

We included all studies that met the following criteria: (1) published as original articles; (2) evaluated human subjects; (3) Tregs were detected in intratumoral tissue, peritumoral tissue, or peripheral blood by testing for markers of CD4+CD25+, FoxP3+ or CD4+CD25+FoxP3+; (4) reported association of high or low Tregs level with overall survival (OS), disease-free survival (DFS), or clinicopathologic parameters; (5) contained the minimum information necessary to estimate the effects (i.e., hazard ratios) and a corresponding measure of uncertainty (i.e., confidence interval, *P*-values, standard errors, or variance). If multiple publications were based on the same patient population, we used the most informative study to avoid duplication. Studies were excluded if they were: (1) reviews or conference abstracts; (2) lacking sufficient data for calculation of incidence and/or hazard ratios (HRs) with 95% confidence intervals (CIs); (3) duplication of previous publications or replicated samples; (4) concerning a rare subtype of Tregs. Two reviewers determined study eligibility independently and disagreements between the reviewers were resolved via discussion and consensus. If they could not reach agreement, a third researcher determined the final results.

### Quality assessment

The quality of the included studies was evaluated according to the Newcastle-Ottawa scale (NOS) criteria for cohort studies[55]. We allocated a score of 0–9 to each included study, and those with a score ≥6 were considered to be of high quality. If disagreement existed on the assigned grade, studies were reassessed until a consensus was reached.

### Data extraction

From each study, the following information was extracted: first author, year of publication, trial design, country, sample size, pretherapy, Tregs marker, Tregs site, Tregs assessment method, cut-off definition, follow-up time, clinicopathologic parameters, and OS or DFS outcome of univariate and/or multivariate analysis (including *P*-values, HRs, and 95% CIs). OS was defined as the interval between curative treatment and death or the last observation for surviving patients. DFS was defined as the interval after curative treatment when no disease can be detected, or from the date of curative treatment to the date of last follow-up for patients without recurrence. If a direct report of survival and recurrence ratios was not available, the survival data from Kaplan–Meier curves were read by Engauge Digitizer version 4.1 (http://digitizer.sourceforge.net/) as described previously [56]. When both univariate analysis and multivariate analysis were reported to obtain the HR, the results of multivariate analysis were selected to avoid confounding factors. Two reviewers performed data extraction using a predefined form. Disagreements were resolved by consensus after discussion.

### Statistical analysis

The overall analysis was performed by assessing all relevant research according to prognostic outcomes and different clinicopathologic parameters. The prognostic effect of the meta-analysis was estimated based on OS and DFS. Effect measures regarding the effect in the meta-analysis were reported as HR with 95% CI. The estimated odds ratio (OR) with 95% CI was used to summarize the correlation between detection of Tregs and clinicopathologic characteristics of hepatocellular carcinoma. Statistical heterogeneity between trials was assessed by the χ2 test and I^2^ statistic [57]. I^2^ values of 25%, 50%, and 75% correspond to cut-off points for low, moderate, and high degrees of heterogeneity. *P* > 0.1 for the χ2 test and I^2^ < 50% were interpreted as signifying low-level heterogeneity. When there was no statistically significant heterogeneity, a pooled effect was calculated with a fixed-effects model; otherwise, a random-effects model was used. The cumulative analysis was performed according to publication time.

Subgroup analyses were carried out to investigate potential sources of heterogeneity between studies and to assess whether conclusions were sensitive to restricting studies to subgroups that might have different prognostic effects. Subgroup analyses were based on therapy, trial design and method of detection. If one study reported an association between Tregs from different tissues and prognosis at the same time, the effect measures based on large sample was used to assess overall prognosis value when ignoring Tregs site, and the effect measure based on small sample was used to analyze the prognostic value of Tregs from different tissues.

Sensitivity analyses were performed to assess the stability of the results; a single study was deleted each time to determine the influence of the individual data set on the results. Publication bias was determined via funnel plot. The *P*-value threshold for statistical significance was set at 0.05 for effect sizes except for the χ^2^ test. The cumulative analysis and sensitivity analyses were conducted with Stata12.0 and other statistical analyses were performed with Review Manager Version 5.3.

## Abbreviations

HCC: hepatocellular carcinoma
Tregs: regulatory T cells
OS: overall survival
DFS: disease-free survival
FoxP3: forkhead or winged helix family of, transcription factor P3

## References

[1] Siegel RL, Miller KD, Jemal A (2015). Cancer statistics, 2015. CA Cancer J Clin.

[2] Jemal A, Bray F, Center MM, Ferlay J, Ward E, Forman D (2011). Global cancer statistics. CA Cancer J Clin.

[3] Sugawara Y (2016). Living donor liver transplantation for patients with hepatocellular carcinoma—20 years after introduction of the Milan criteria. HepatoBiliary Surg Nutr.

[4] Li S, Yang F, Ren X (2015). Immunotherapy for hepatocellular carcinoma. Drug Discov Ther.

[5] Weiskirchen R, Tacke F (2016). Immune surveillance of liver cancer in non-alcoholic fatty liver disease: excess lipids cause CD4 T-cells loss and promote hepatocellular carcinoma development. HepatoBiliary Surg Nutr.

[6] Nishida N, Kudo M (2017). Immunological microenvironment of hepatocellular carcinoma and its clinical implication. Oncology.

[7] Hori S, Nomura T, Sakaguchi S (2003). Control of regulatory T cell development by the transcription factor Foxp3. Science.

[8] Curiel TJ, Coukos G, Zou L, Alvarez X, Cheng P, Mottram P, Evdemon-Hogan M, Conejo-Garcia JR, Zhang L, Burow M (2004). Specific recruitment of regulatory T cells in ovarian carcinoma fosters immune privilege and predicts reduced survival. Nat Med.

[9] Oleinika K, Nibbs R, Graham G, Fraser A (2013). Suppression, subversion and escape: the role of regulatory T cells in cancer progression. Clin Exp Immunol.

[10] Pedroza-Gonzalez A, Verhoef C, Ijzermans JN, Peppelenbosch MP, Kwekkeboom J, Verheij J, Janssen HL, Sprengers D (2013). Activated tumor-infiltrating CD4+ regulatory T cells restrain antitumor immunity in patients with primary or metastatic liver cancer. Hepatology.

[11] Guo CL, Yang XH, Cheng W, Xu Y, Li JB, Sun YX, Bi YM, Zhang L, Wang QC (2014). Expression of Fas/FasL in CD8+ T, CD3+ Foxp3+ Treg cells—Relationship with apoptosis of circulating CD8+ T cells in hepatocellular carcinoma patients. Asian Pac J Cancer Prev.

[12] Morse MA, Hobeika AC, Osada T, Serra D, Niedzwiecki D, Lyerly HK, Clay TM (2008). Depletion of human regulatory T cells specifically enhances antigen-specific immune responses to cancer vaccines. Blood.

[13] Zhou S, Tao H, Zhen Z, Chen H, Chen G, Yang Y (2013). Depletion of CD4+ CD25+ regulatory T cells promotes CCL21-mediated antitumor immunity. PLoS One.

[14] Unitt E, Marshall A, Gelson W, Rushbrook SM, Davies S, Vowler SL, Morris LS, Coleman N, Alexander GJ (2006). Tumour lymphocytic infiltrate and recurrence of hepatocellular carcinoma following liver transplantation. J Hepatol.

[15] Fu J, Xu D, Liu Z, Shi M, Zhao P, Fu B, Zhang Z, Yang H, Zhang H, Zhou C (2007). Increased regulatory T cells correlate with CD8 T-cell impairment and poor survival in hepatocellular carcinoma patients. Gastroenterology.

[16] Kobayashi N, Hiraoka N, Yamagami W, Ojima H, Kanai Y, Kosuge T, Nakajima A, Hirohashi S (2007). regulatory T cells affect the development and progression of hepatocarcinogenesis. Clin Cancer Res.

[17] Huang Y, Liao H, Zhang Y, Yuan R, Wang F, Gao Y, Wang P, Du Z (2014). Prognostic value of tumor-infiltrating FoxP3+ T cells in gastrointestinal cancers: a meta analysis. PLoS One.

[18] Shang B, Liu Y, Jiang Sj, Liu Y (2015). Prognostic value of tumor-infiltrating FoxP3+ regulatory T cells in cancers: a systematic review and meta-analysis. Sci Rep.

[19] Gao Q, Qiu SJ, Fan J, Zhou J, Wang XY, Xiao YS, Xu Y, Li YW, Tang ZY (2007). Intratumoral balance of regulatory and cytotoxic T cells is associated with prognosis of hepatocellular carcinoma after resection. J Clin Oncol.

[20] Sasaki A, Tanaka F, Mimori K, Inoue H, Kai S, Shibata K, Ohta M, Kitano S, Mori M (2008). Prognostic value of tumor-infiltrating FOXP3+ regulatory T cells in patients with hepatocellular carcinoma. Eur J Surg Oncol.

[21] Gao Q, Wang XY, Qiu SJ, Yamato I, Sho M, Nakajima Y, Zhou J, Li BZ, Shi YH, Xiao YS, Xu Y, Fan J (2009). Overexpression of PD-L1 significantly associates with tumor aggressiveness and postoperative recurrence in human hepatocellular carcinoma. Clin Cancer Res.

[22] Ju MJ, Qiu SJ, Gao Q, Fan J, Cai MY, Li YW, Tang ZY (2009). Combination of peritumoral mast cells and T-regulatory cells predicts prognosis of hepatocellular carcinoma. Cancer Sci.

[23] Zhou J, Ding T, Pan W, Zhu LY, Li L, Zheng L (2009). Increased intratumoral regulatory T cells are related to intratumoral macrophages and poor prognosis in hepatocellular carcinoma patients. Int J Cancer.

[24] Ju MJ, Qiu SJ, Fan J, Xiao YS, Gao Q, Zhou J, Li YW, Tang ZY (2009). Peritumoral activated hepatic stellate cells predict poor clinical outcome in hepatocellular carcinoma after curative resection. Am J Clin Pathol.

[25] Wang WH, Jiang CL, Yan W, Zhang YH, Yang JT, Zhang C, Yan B, Zhang W, Han W, Wang JZ (2010). FOXP3 expression and clinical characteristics of hepatocellular carcinoma. World J Gastroenterol.

[26] Lin G, Wang J, Li S, Xu L, Li S (2010). Relationship and clinical significance of TGF-beta1 expression with Treg cell infiltration in hepatocellular carcinoma. Chin J Cancer.

[27] Zhou L, Fu JL, Lu YY, Fu BY, Wang CP, An LJ, Wang XZ, Zeng Z, Zhou CB, Yang YP (2010). Regulatory T cells are associated with post-cryoablation prognosis in patients with hepatitis B virus-related hepatocellular carcinoma. J Gastroenterol.

[28] Chen KJ, Lin SZ, Zhou L, Xie HY, Zhou WH, Taki-Eldin A, Zheng SS (2011). Selective recruitment of regulatory T cell through CCR6-CCL20 in hepatocellular carcinoma fosters tumor progression and predicts poor prognosis. PLoS One.

[29] Shen SL, Liang LJ, Peng BG, He Q, Kuang M, Lai JM (2011). Foxp3+ regulatory T cells and the formation of portal vein tumour thrombus in patients with hepatocellular carcinoma. Can J Surg.

[30] Chen KJ, Zhou L, Xie HY, Ahmed TE, Feng XW, Zheng SS (2012). Intratumoral regulatory T cells alone or in combination with cytotoxic T cells predict prognosis of hepatocellular carcinoma after resection. Med Oncol.

[31] Li HZ, Guo Z, Wang HT, Si TG, Liu CF, Yu HP (2012). The effect of regulatory T cells level in peripheral blood on the prognosis in HCC patients after TACE. Journal of Interventional Radiology.

[32] Huang Y, Wang FM, Wang T, Wang YJ, Zhu ZY, Gao YT, Du Z (2012). [Tumor-infiltrating FoxP3+ Tregs are associated with CD34 expression and prognosis of hepatocellular carcinoma]. [Article in Chinese]. Zhonghua Gan Zang Bing Za Zhi.

[33] Wang F, Jing X, Li G, Wang T, Yang B, Zhu Z, Gao Y, Zhang Q, Yang Y, Wang Y (2012). Foxp3+ regulatory T cells are associated with the natural history of chronic hepatitis B and poor prognosis of hepatocellular carcinoma. Liver International.

[34] Lin SZ, Chen K, Xu ZY, Chen H, Xie Hy, Zhou L, Zheng SS (2013). Prediction of recurrence and survival in hepatocellular carcinoma based on two Cox models mainly determined by FoxP3+ regulatory T cells. Cancer Prev Res (Phila).

[35] Huang Y, Wang F, Wang Y, Zhu Z, Gao Y, Ma Z, Xu R, Du Z (2014). Intrahepatic interleukin-17+ T cells and FoxP3+ regulatory T cells cooperate to promote development and affect the prognosis of hepatocellular carcinoma. J Gastroenterol Hepatol.

[36] Li F, Guo Z, Lizée G, Yu H, Wang H, Si T (2014). Clinical prognostic value of CD4+ CD25+ FOXP3+ regulatory T cells in peripheral blood of Barcelona Clinic Liver Cancer (BCLC) stage B hepatocellular carcinoma patients. Clin Chem Lab Med.

[37] Zhou Y, Wang B, Wu J, Zhang C, Zhou Y, Yang X, Zhou J, Guo W, Fan J (2016). Association of preoperative EpCAM Circulating Tumor Cells and peripheral Treg cell levels with early recurrence of hepatocellular carcinoma following radical hepatic resection. BMC Cancer.

[38] Wang Y, Liu T, Tang W, Deng B, Chen Y, Zhu J, Shen X (2016). Hepatocellular Carcinoma Cells Induce Regulatory T Cells and Lead to Poor Prognosis via Production of Transforming Growth Factor-β1. Cell Physiol Biochem.

[39] Wang Q, Luan W, Warren L, Fiel MI, Blank S, Kadri H, Mandeli J, Hiotis SP (2016). Prognostic Role of Immune Cells in Hepatitis B-associated Hepatocellular Carcinoma Following Surgical Resection Depends on Their Localization and Tumor Size. J Immunother.

[40] Tu JF, Ding YH, Ying XH, Wu FZ, Zhou XM, Zhang DK, Zou H, Ji JS (2016). Regulatory T cells, especially ICOS+ FOXP3+ regulatory T cells, are increased in the hepatocellular carcinoma microenvironment and predict reduced survival. Sci Rep.

[41] Fu YP, Yi Y, Cai XY, Sun J, Ni XC, He HW, Wang JX, Lu ZF, Huang JL, Cao Y (2016). Overexpression of interleukin-35 associates with hepatocellular carcinoma aggressiveness and recurrence after curative resection. Br J Cancer.

[42] Cai XY, Ni XC, Yi Y, He HW, Wang JX, Fu YP, Sun J, Zhou J, Cheng YF, Jin JJ (2016). Overexpression of CD39 in hepatocellular carcinoma is an independent indicator of poor outcome after radical resection. Medicine.

[43] Jiang D, Gao Z, Cai Z, Wang M, He J (2015). Clinicopathological and prognostic significance of FOXP3+ tumor infiltrating lymphocytes in patients with breast cancer: a meta-analysis. BMC Cancer.

[44] Salama P, Phillips M, Grieu F, Morris M, Zeps N, Joseph D, Platell C, Iacopetta B (2009). Tumor-infiltrating FOXP3+ T regulatory cells show strong prognostic significance in colorectal cancer. J Clin Oncol.

[45] Tai X, Van Laethem F, Pobezinsky L, Guinter T, Sharrow SO, Adams A, Granger L, Kruhlak M, Lindsten T, Thompson CB (2012). Basis of CTLA-4 function in regulatory and conventional CD4+ T cells. Blood.

[46] Marie JC, Letterio JJ, Gavin M, Rudensky AY (2005). TGF-β1 maintains suppressor function and Foxp3 expression in CD4+ CD25+ regulatory T cells. J Exp Med.

[47] Cobbold SP, Adams E, Farquhar CA, Nolan KF, Howie D, Lui KO, Fairchild PJ, Mellor AL, Ron D, Waldmann H Infectious tolerance via the consumption of essential amino acids and mTOR signaling. Proceedings of the National Academy of Sciences.

[48] Erhardt A, Biburger M, Papadopoulos T, Tiegs G (2007). IL-10, regulatory T cells, and Kupffer cells mediate tolerance in concanavalin A–induced liver injury in mice. Hepatology.

[49] Wu H, Chen P, Liao R, Li YW, Yi Y, Wang JX, Cai XY, He HW, Jin JJ, Cheng YF (2013). Intratumoral regulatory T cells with higher prevalence and more suppressive activity in hepatocellular carcinoma patients. J Gastroenterol Hepatol.

[50] Miyara M, Yoshioka Y, Kitoh A, Shima T, Wing K, Niwa A, Parizot C, Taflin C, Heike T, Valeyre D (2009). Functional delineation and differentiation dynamics of human CD4+ T cells expressing the FoxP3 transcription factor. Immunity.

[51] Mrizak D, Martin N, Barjon C, Jimenez-Pailhes AS, Mustapha R, Niki T, Guigay J, Pancré V, de Launoit Y, Busson P (2015). Effect of nasopharyngeal carcinoma-derived exosomes on human regulatory T cells. J Natl Cancer Inst.

[52] Zhao HQ, Li WM, Lu ZQ, Yao YM (2014). Roles of Tregs in development of hepatocellular carcinoma: a meta-analysis. World J Gastroenterol.

[53] Wu H, Chen P, Liao R, Li YW, Yi Y, Wang JX, Sun TW, Zhou J, Shi YH, Yang XR (2012). Overexpression of galectin-1 is associated with poor prognosis in human hepatocellular carcinoma following resection. J Gastroenterol Hepatol.

[54] Liberati A, Altman DG, Tetzlaff J, Mulrow C, Gøtzsche PC, Ioannidis JP, Clarke M, Devereaux PJ, Kleijnen J, Moher D (2009). The PRISMA statement for reporting systematic reviews and meta-analyses of studies that evaluate health care interventions: explanation and elaboration. PLoS Med.

[55] Stang A (2010). Critical evaluation of the Newcastle-Ottawa scale for the assessment of the quality of nonrandomized studies in meta-analyses. Eur J Epidemiol.

[56] Tierney JF, Stewart LA, Ghersi D, Burdett S, Sydes MR (2007). Practical methods for incorporating summary time-to-event data into meta-analysis. Trials.

[57] Higgins J, Thompson SG (2002). Quantifying heterogeneity in a meta-analysis. Stat Med.

